# Influence of bruxism on survival of porcelain laminate veneers

**DOI:** 10.4317/medoral.19097

**Published:** 2013-08-29

**Authors:** Maria Granell-Ruíz, Rubén Agustín-Panadero, Antonio Fons-Font, Juan L. Román-Rodríguez, María F. Solá-Ruíz

**Affiliations:** 1 DDS,PhD. Associate Profesor, Oclusion and Prosthodontics, Department of Stomatology, University of Valencia, Valencia, Spain; 2 DDS,PhD,MD. Professor of Oclusion and Prosthodontics, Department of Stomatology, University of Valencia, Valencia, Spain; 3 MF,DDS,PhD,MD. Adjunct Lecturer, Department of Stomatology, University of Valencia, Valencia, Spain

## Abstract

Objectives: This study aims to determine whether bruxism and the use of occlusal splints affect the survival of porcelain laminate veneers in patients treated with this technique. Material and Methods: Restorations were made in 70 patients, including 30 patients with some type of parafunctional habit. A total of 323 veneers were placed, 170 in patients with bruxism activity, and the remaining 153 in patients without it. A clinical examination determined the presence or absence of ceramic failure (cracks, fractures and debonding) of the restorations; these incidents were analyzed for association with bruxism and the use of splints. Results: Analysis of the ceramic failures showed that of the 13 fractures and 29 debonding that were present in our study, 8 fractures and 22 debonding were related to the presence of bruxism. Conclusions: Porcelain laminate veneers are a predictable treatment option that provides excellent results, recognizing a higher risk of failure in patients with bruxism activity. The use of occlusal splints reduces the risk of fractures.

** Key words:**Veneer, fracture, debonding, bruxism, occlusal splint.

## Introduction

The veneer restoration technique was developed in the mid nineteen-eighties in the United States, and later spread throughout the world. Bonding these fragile porcelain laminae securely to natural teeth has been a challenge for our profession. Fortunately, these restorations have proven to be one of the most successful techniques used in Restorative Dentistry (1).

Porcelain laminate veneers represent a predictable restorative solution for anterior teeth due to their excellent aesthetics as well as their durability and biocompatibi-lity (2). These restorations constitute an alternative to full-coverage restorations since they require minimal tooth preparation, there by maintaining the dental structure.

Currently, porcelain laminate veneers are indicated for a wide range of situations and can be used to correct the shape and position of teeth, close diastema, replace old composite restorations, mask tooth discoloration (3), and to restore teeth following incisal abrasion and dental erosion. Some authors (4,5) suggest that bruxism constitutes a contraindication to these bonded restorations. Bruxism is generally recognized as non-functional jaw movements, and is defined as a forcible clenching or grinding of the teeth, or a combination of both, and has long been regarded as a disorder requiring treatment (6). According to the American Academy of Orofacial Pain, bruxism is a diurnal or nocturnal parafunctional activity which includes clenching, bracing, gnashing and grinding of the teeth (7). Magne et al. report that the success rate for the veneer is reduced to 60% in patients with bruxism activity (8). This percentage is very similar to that obtained for metal-ceramic restorations in the same situation. The success rates may be increased if bruxism iscontrolled; therefore, a nocturnal and / or diurnal splint is recommended as a preventive measure to reduce the risk of failure, especially in these patients (4,9).

The occlusal splint is generally used to treat muscle hyperactivity. Studies carried out by various authors (10-13) show that these splints decrease bruxism activity generated during periods of stress; it is therefore advisable to use these devices in patients with suspected bruxism following prosthodontic treatment with either full coverage crowns or with laminate veneers.

Restorations placed in patients presenting some type of bruxism activity should have a functional design, especially in situations where the patient has already lost some tooth structure and where these restorations provide the patient with a correct anterior and canine guidance (14).

As with any technique, the use of porcelain veneers requires medium and long term studies to confirm their indications (4,9,15-20).

These techniques have been used since 1985 at the Prosthodontics and Oclussion Teaching Unit of the University of Valencia, School of Medicine and Dentistry, where to date a large number of patients have been treated with porcelain laminate veneers in response to aesthetic demands.

We conducted a retrospective clinical study to review patients wearing porcelain laminate veneers. We analyzed whether the presence of bruxims activity and the use of occlusal splints in our patients, affected the medium and long term survival of these treatments. To this end we developed a data collection methodology to provide reliable results able to withstand the usual statistical tests for these sample types and to be compared with results of other authors.

## Material and Methods

Three hundred twenty-three porcelain laminate veneers were placed during a period of eight years, all fabricated with IPS-Empress ceramic (Ivoclar®, Schaan, Liechtenstein) in order to standardize the results and eliminate any variables that could arise from the use of different ceramics.

At the time of the study, the 323 restorations studied had been placed in 70 patients with a duration ranging from 3 to 11 years. Of the patients studied, 24.3% (17) were male and 75.7% (53) were female, with a mean age of 46 years (range 18 to 74). Thirty of the 70 patients presented bruxism activity, all patients with it, had to use occlusal splints (Hard acrylic), 15 complied with this requirement and 15 did not. The clinical diagnosis was made by clinical inspection of teeth of the consequences of clenching or grinding activities were visible in the dentition and consistent with a bruxing habit.

Of the 323 veneers, 124 (38.4%) were of simple design or window preparation, covering only the buccal surface (B) and 199 (61.6%) corresponded to those denominated ‘functional’ (with incisal overlap), covering the incisal edge and part of the palatal/lingual tooth surface (F). Regarding location, 238 were placed in the maxillary arch and 85 in the mandibular arch. Of the maxillary restorations, 97 were on central incisors, 82 on lateral incisors, 49 on canines and 10 on premolars. Of the mandibular restorations, 31 were located on central incisors, 31 on lateral incisors, 19 on canines and 4 on premolars.

One hundred seventy veneers were bonded in patients with bruxism activity and 153 in patients without it.

This study focussed on the relationship between the different ceramic failures and bruxism; therefore information was collected on the presence or absence of bruxism activity and whether or not these patients had to use splints. These criteria provided us with 3 patients groups for the study:

A- Patients without bruxism. This group included 40 patients, representing 57.1% of the total. These patients were restored with 153 veneers (65 conventional design and 88 functional).

B- Patients with bruxism activity using splints properly. This group included 15 patients (21.4%) with 89 veneers (31 conventional and 58 functional).

C- Patients with bruxism activity not using splints (they have it but they don´t use it). This group included 15 patients (21.4%) with 81 veneers (28 conventional and 53 functional).

Therefore, after placing the ceramic restorations, we checked occlusion properly, during maximum intercuspation and during mandibular excursive movements. Patients who were bruxers were provided with hard acrylic resin occlusal guards to protect the definitive restorations during bruxing episodes.

All of the patients were treated at the Prosthodontics and Occlusion Teaching Unit of the University of Valencia School of Medicine and Dentistry by a team that had followed the same method when placing the veneers.

The statistical analysis focused on:

An initial descriptive analysis containing the frequencies and percentages for the categorical variables in the study.

A bivariate analysis, covering all the statistical comparisons necessary to assess the relationship between fractures and debonding in patients with bruxism activity, and the use of a splint by these patients. These analyses were performed using nonparametric statistical tests given the categorical nature of the variables.

The Pearson c2 test was used to test the association or dependence between two categorical variables, always provided that more than 5 cases were present in the contingency tables. Otherwise, and only for dichotomous variables, the Fisher’s exact test was used.

A Kaplan-Meier Survival Analysis was used to study survival. As a comparative test the log-rank test was used (Kaplan-Meier, 1958).

## Results

During the evaluation period, the results were:

Ceramic failures: The survival of restorations in terms of their structural integrity is the most important factor for both patients and professionals when deciding on this treatment option. Therefore, the analysis was made in terms of the presence or absence of the three most important aspects: cracks, fractures and debonding ( Table 1).

**Table 1:**
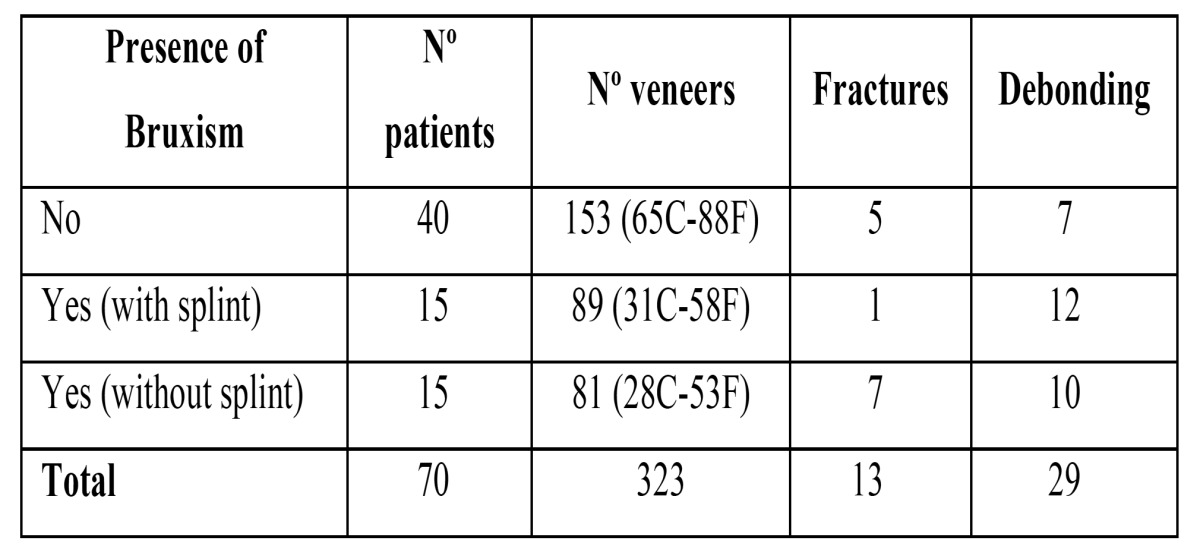
Distribution of veneers restorations. Frequency of fractures and debonding.

 Cracks: At the time of the review no cracks were observed. This does not mean that some of the fractures found had not initiated as a crack, which over time had developed into a fracture.

 Fractures: A total of 13 fractures were observed (4%). Eight appeared in patients with bruxism, and the remaining 5 in patients without it.

 Debonding: A total of 29 debonded restorations were observed, corresponding to 9% of the sample. Twenty-two were found in patients with bruxism, and the remaining 7 in patients without it.

**Figure 1:**
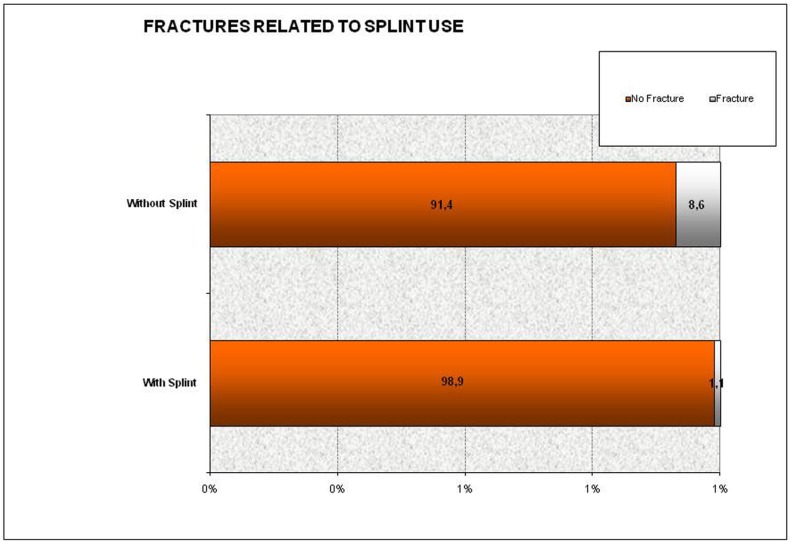
Percentage of veneers fractures and use of splint.

By statistically relating ceramic failures with bruxism, a clear link can be seen. On one hand we can see that fractures, although more frequent in the presence of bruxism, are not statistically significant, given that 5 fractures appeared in patients without bruxism versus 8 fractures that occurred in patients with it (*p* = 0.511) (Chi2); in contrast, statistically significant differences were found when examining the correct use of splints in patients with bruxism, since of these 8 fractures, 1 occurred in a patient who did use a splint, and 7 in patients who did not (*p* = 0.023) (Fisher). The figure below shows that a higher proportion of fractures were observed in patients with bruxism activity who did not use a splint (9%) than in those who used a splint pro-perly (1%) (Fig. 1).

**Figure 2:**
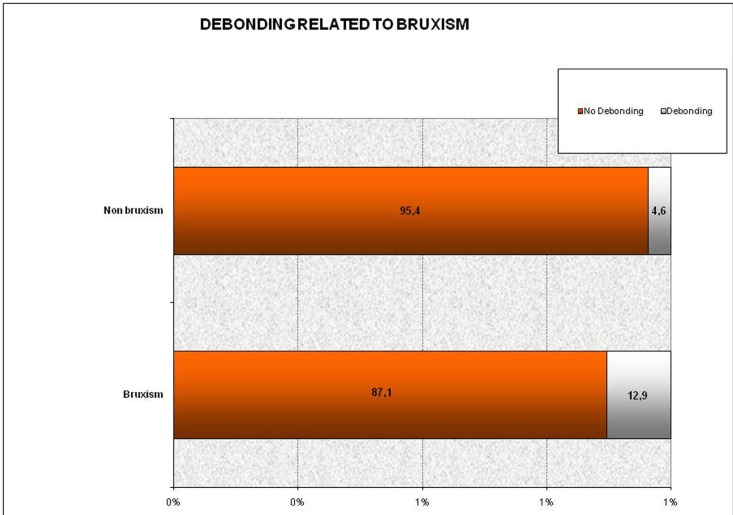
Percentage of veneers debonding and patients with bruxism.

Regarding debonding, this was observed to be more frequent in patients with bruxism. Of the 29 debonded veneers, 22 were produced in these patients (*p* = 0.009) (Chi2), a clear statistically significant difference can be seen between the two groups of patients (with and without bruxism activity). The figure below illustrates the higher proportion of debonding in patients with bruxism versus those without it (Fig. 2). Of the 22 debonded restorations in patients with bruxism, 12 appeared in patients using a splint and 10 in patients where splints were not used, without statistically significant differences (*p* = 0.825) (Chi2).

Regarding design, there were no significant differences between the type of restoration used (conventional or functional) and the presence of bruxism activity (*p* = 0.151) (Chi2); although, in this study most patients with bruxism were fitted with functional restorations (F).

The Kaplan-Meier curves (Kaplan-Meier, 1958) clearly show the survival of the restorations, indicating the probability that a restoration will remain in good condition over time.

This analysis considered the time in years during which the restoration remained in good condition or the time until deterioration. Two types of deterioration were considered: Debonding and fracture, in addition this deterioration was related to the presence or absence of bruxism.

**Figure 3:**
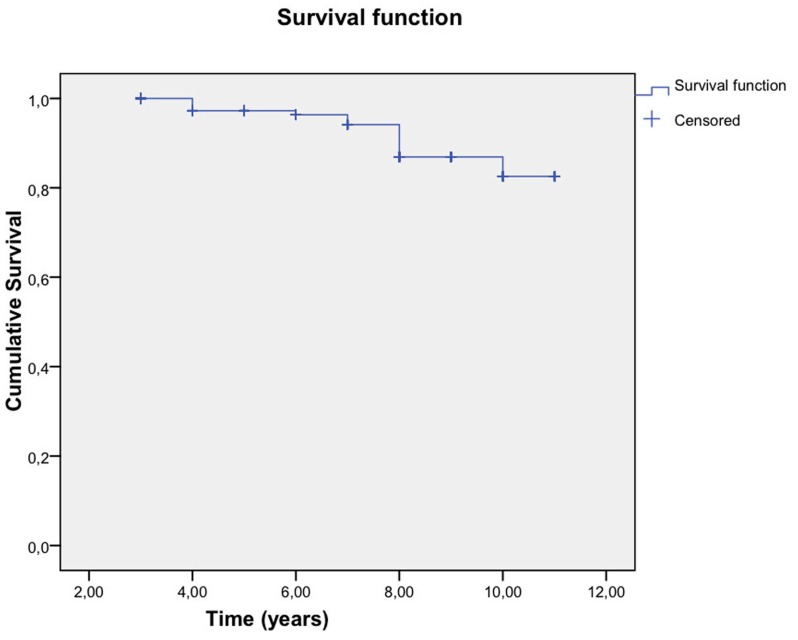
Survival estimates according veneers debonding.

Fractures: The estimated survival table showed that the mean survival times were similar between patients with and without bruxism. Furthermore, the log-rank test confirmed that the survival curves were statistically equal (*p* = 0.519) (Fig. 3).

**Figure 4:**
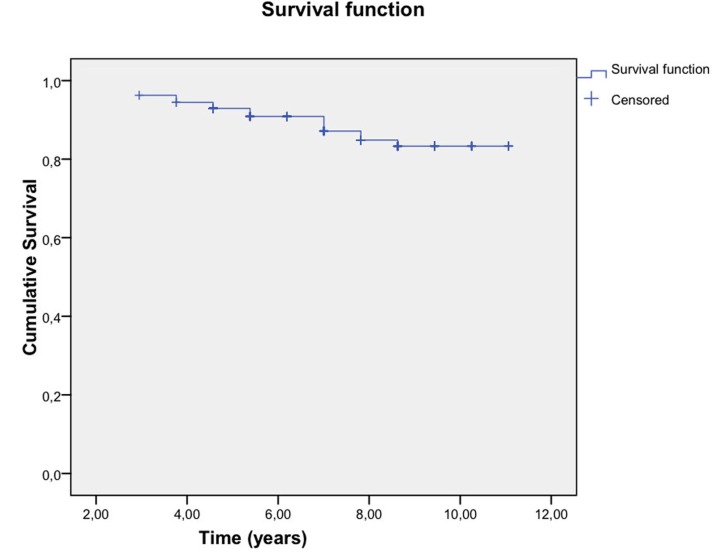
Survival estimates according veneers fractures.

Debonding: Although the estimated survival table indicated that the mean survival times were similar between patients with and without bruxism, the log-rank test confirmed statistically significant differences in the survival curves (*p* = 0.008) (Fig. 4).

## Discussion

To date many longitudinal clinical studies have investigated the performance of porcelain veneers (4,9,15-20).

It has been shown that clinical studies are needed in order to evaluate the performance of restorative materials, given that certain intraoral conditions cannot be duplicated in the laboratory. These situations include the application of multiple, intermittent and cyclical forces on biting, chewing or grinding; the constant exposure to a moist, bacteria-rich environment; the consumption of hot and cold liquids, as well as vigorous brushing. In vivo studies are therefore necessary to verify the acceptability of a laminate veneer as a definitive restorative treatment. Retrospective studies can provide a reliable picture of the clinical performance of materials and techniques.

While numerous in vitro studies exist (21,22), these do not offer the same prognostic value or long-term predictability of this treatment as studies in vivo. Although longitudinal clinical studies of longer than 5 years certainly provide useful scientific data, they can sometimes become out of date due to the rapid and constant change in technology and materials. Thus, in vitro studies may have more impact, but have no greater utility.

Discussion of results

With respect to ceramic failures, in the present study there were 13 fractures (4.0%), 29 debondings (9.0%) and no cracks.

Cracks: The fact that in this study no cracks appeared in the restorations may be due to the use of high-strength porcelain (IPS-Empress). Magne *et al*. (8) in a clinical study used conventional feldspathic porcelain for the fabrication of the restorations; the authors observed 12% cracks, thus justifying the use of stronger porcelain. The majority of authors do not consider small cracks in restorations as failures (5,23).

Fractures: We found 4% of fractures, data similar to those of Jordan *et al*. (15) and Calamia (24) with 3%, and Nordbø *et al*. (17) with 5%. The majority of clinical studies reviewed report a low incidence of fractures, for example Kinh *et al*. (23) 0%; and Peumans *et al*. (5) 1%. However, other authors indicate a much higher rate of fractures, Christensen *et al*. (9) reported 13% at 3 years and Walls (4) 14% at 5 years, arguing that the majority of their patients had a history of bruxism, and that they had used conventional feldspathic porcelain, which has a lower fracture strength than high-strength feldspathic restorations.

In the present study it was observed that fractures occurred more frequently in patients with bruxism, and that not using a splint when required constitutes a risk factor for the presence of fractures.

Debonding: there was a notably high percentage of debonding in this study (9%), a high proportion of which occurred in patients with bruxism. It was found that of the 22 debonded veneers in patients with bruxism, 12 were related to patients who used an occlusal splint, while the remaining 10 were related to patients who did not use a splint; we therefore consider that the debonding was not so much related to the use or otherwise of a splint, but more to the existence of a history of bruxism, taking into account that these patients generally wear the splint only at night, and it has been found that bruxism may be both diurnal and nocturnal (25).

Some authors (16,18) with in vivo studies report high rates of debonding in restorations due to the presence of composite reconstructions in teeth supporting this type of restoration. In these cases the adhesion is between resin and resin, which reduces the bond strength between the porcelain veneer-tooth complexes.

Some authors do not consider debonding as a failure, since the restoration is simply replaced. Fradeani *et al*. (19), report three cases of debonding in one of their studies, with no signs of internal damage or fracture; these were replaced, commenting that the debonding was most certainly due to an inappropriate adhesive technique.

## Conclusions

1. In this study, the presence of fractures and debonding in porcelain laminate veneers increases considerably in patients with bruxism. The probability of debonding is almost 3 times higher in patients with it.

2. It was found that, the use of splints reduces the failure rate of porcelain laminate veneers in patients with bruxism activity; the probability of fracture being 8 times greater in patients who are required to use a splint but do not.

3. Longitudinal in vivo clinical studies are needed to evaluate the performance and predictability of restorative materials, since certain intraoral conditions cannot be reproduced in the laboratory.
